# The Use of Nitrogen and Its Regulation in Cereals: Structural Genes, Transcription Factors, and the Role of miRNAs

**DOI:** 10.3390/plants8080294

**Published:** 2019-08-20

**Authors:** Diana L. Zuluaga, Gabriella Sonnante

**Affiliations:** Institute of Biosciences and Bioresources, National Research Council, Via Amendola 165/A, 70126 Bari, Italy

**Keywords:** nitrogen use, cereals, miRNA, target genes, nitrogen assimilation, nitrogen transport, nitrogen remobilization

## Abstract

Cereals and, especially, rice, maize, and wheat, are essential commodities, on which human nutrition is based. Expanding population and food demand have required higher production which has been achieved by increasing fertilization, and especially nitrogen supply to cereal crops. In fact, nitrogen is a crucial nutrient for the plant, but excessive use poses serious environmental and health issues. Therefore, increasing nitrogen use efficiency in cereals is of pivotal importance for sustainable agriculture. The main steps in the use of nitrogen are uptake and transport, reduction and assimilation, and translocation and remobilization. Many studies have been carried out on the genes involved in these phases, and on transcription factors regulating these genes. Lately, increasing attention has been paid to miRNAs responding to abiotic stress, including nutrient deficiency. Many miRNAs have been found to regulate transcription factors acting on the expression of specific genes for nitrogen uptake or remobilization. Recent studies on gene regulatory networks have also demonstrated that miRNAs can interact with several nodes in the network, functioning as key regulators in nitrogen metabolism.

## 1. Introduction

Cereal crops are cultivated in larger quantities than any other crop in the world. Since the Green Revolution started, the production of high-yielding cereal crops has significantly increased. Maize (*Zea mays* L.), common wheat (*Triticum aestivum* L.), and rice (*Oryza sativa* L.) are the most important crops for human nutrition, representing about 90% of all cereal production worldwide. These three crops provide the majority of proteins and calories consumed by humans both directly with the grain, or indirectly through livestock products [1,2,3].

Plants depend on inorganic nitrogen (N) availability, with this element being essential for the composition of vital plant molecules such as nucleic acids, chlorophyll, and proteins. Due to the high importance of N for high-yield agriculture, more than 100 TgNyr^−1^ of reactive N is produced industrially using fossil fuels as energy sources and is applied worldwide to crop fields [1]. From this large amount, 50% is used to fertilize maize, wheat, and rice. Nitrogen is the most important mineral nutrient for cereal crops since high yields depend on an adequate N supply, and cereal seeds contain storage protein reserves with about 6% N [1,4]. Farmers, therefore, apply about 80 million tons of N fertilizers to cereal crops every year worldwide, however, less than 40% of the N added to cereal fields by means of fertilizers is taken up by the crops, while the remaining part is dispersed in the environment, causing water pollution and releasing greenhouse gases [5], thus affecting climate change. Moreover, nitrate production expends up to 2% of the world energy, and therefore has consequences also on the carbon balance [6]. In order to reduce the impact of agriculture on climate change and, at the same time, to manage the sustainable feeding of a growing world population, improving nitrogen use efficiency (NUE) in cereals is, and should continue to be, a priority for crop breeders.

Micro RNAs (miRNAs) are a class of small RNA molecules, 20–24 nucleotides in length, widely distributed in living organisms and highly evolutionarily conserved in plants [7], substantiating the findings that miRNAs are involved in important common traits (e.g., plant morphology). However, especially during the last decade, with the advent of massive sequencing, many non-conserved, species-specific miRNAs have been discovered, which may control variable processes in different plant species. MiRNAs regulate cell signaling mechanisms in plants by the attenuation of gene expression at the post-transcriptional level or via translational inhibition [8]. Both conserved and specific miRNAs can be powerful regulators of plant growth, development, and adaptation to biotic and abiotic environmental stimuli [9,10]. The role of miRNAs in plant abiotic stress responses, including nutrient deficiency, is widely known [11]. These small RNA molecules are implicated in nutrient uptake, transport, and assimilation and are able to move between cells and through the vascular system, being signaling molecules between cells, tissues, and organs [12]. Among other nutrients, miRNAs also regulate the use of nitrogen in crop plants, and therefore participate in the adaptation of crops to nitrogen deficiency.

In this review, the authors summarize what has been discovered in the last 20 years regarding the use of nitrogen in cereals, with a special focus on miRNAs involved in gene regulation. This information can be used in cereal breeding programs addressing the selection of varieties with a higher NUE, and consequently higher and more sustainable productivity.

## 2. The Use of Nitrogen in Cereals

The use of N in plants involves a number of phases, mainly N uptake, reduction, assimilation, translocation, and remobilization [13]. Nitrogen use efficiency in cereals is defined as the grain yield per unit of N available in the soil [14]. Cereal NUE is the result between plant uptake efficiency (NUpE) and the utilization efficiency (NUtE), and therefore the combination between how effectively plants capture N, and how plants use the N that is taken up [15]. Genetic differences in N uptake or grain production per unit of N applied have been studied in the economically important Graminaceae crops, including wheat, rice, maize, sorghum, and barley [16,17,18,19,20].

Here we describe the main steps of the use of N in cereal crops.

### 2.1. Nitrogen Uptake and Transport

Soil nitrogen availability is usually low, but it can be variable depending on pedoclimatic factors such as precipitation, temperature, wind, pH, and soil type [13]. The form of nitrogen preferred by crops depends on crop adaptation to soil conditions. Nitrate (NO_3_^−^) is the major source of N in agricultural soils and the predominant N form available for cereal crops [5]. However, NO_3_^−^ concentrations in soils can be very variable, and therefore crops have developed specific adaptations to absorb the available NO_3_^−^. A high affinity transport system (HATS) and a low affinity transport system (LATS), placed in the cell membrane of the roots, are active in low and high NO_3_^−^ conditions, respectively. Nitrate is actively transported mainly via members of the NRT families of NO_3_^−^ transporters. In Arabidopsis, these transporters belong to the following three main families: the NRT1 family, whose members are predominantly low-affinity transporters, and the NRT2/NRT3 (NAR2) proteins, which play an important role in the NO_3_^−^ high-affinity transport [21]. Using a reciprocal best hit (RBH) approach, putative orthologues of Arabidopsis *NRT* genes have been identified in maize, rice, sorghum, and *Brachypodium distachyon*, revealing important differences in gene number and family structure [21]. In contrast to Arabidopsis, grasses display an additional *NRT1.1* orthologue and lack of *NRT1.6*/*NRT1.7* orthologues. The *NRT2* genes in grasses show a significant genetic distance, implying that proper functional analysis in cereals should be done to explore *NRT2* function [21,22]. A recent study in the monocot model plant *B. distachyon* identified seven genes encoding putative high-affinity nitrate transporters (BdNRT2), demonstrating that genes in the BdNRT2 family have diverse roles, in response to various N conditions, different from the AtNRT2 family in Arabidopsis. Moreover, it has been shown that *BdNRT2.1* serves as a key member of the family and is strongly induced by nitrate resupply together with *BdNRT2.2*, while the *BdNRT2.5* gene is repressed and other genes are constitutively expressed in roots [22]. In rice, there are four high affinity NTR2 [23]. *OsNRT2.1* and *Os-NRT2.2* genes are highly similar to the corresponding homologues in other monocotyledonous plants, while *OsNRT2.3* and *OsNRT2.4* are closely related to Arabidopsis NRT2 [24]. Recently, it was demonstrated that overexpression of the nitrate transporter gene *OsNRT2.3b* significantly improves grain yield and NUE in rice (Table 1) by enhancing the pH-buffering capacity [25]. In common wheat, sixteen low-affinity nitrate transporter *NPF* (corresponding to NRT1) genes, homologous to *Arabidopsis NPFs,* have been identified [26]. The *NPF* wheat genes have been revealed to be regulated by plant N status, suggesting involvement of these transporters in substrate transport with regard to N metabolism.

Although N in agricultural soils is predominantly available as NO_3_^−^, in some cases, such as for rice plants grown in paddy fields, ammonium ions (NH_4_^+^) are the major source of inorganic nitrogen [27]. Physiological studies have demonstrated the presence of one HATS for NH_4_^+^ in roots of higher plants constituted by a set of ammonium transporter/methylamine permease/Rhesus-type (AMT/MEP/Rh-type) protein family, responsible for ammonium transport. In the roots of *A. thaliana*, five AMT-type transporters are expressed, all of them becoming up-regulated under N-deficient environments. In this species, most of the high affinity uptake capacity of NH_4_^+^ is mediated by AMT1-1, AMT1-2, and AMT1-3 [28,29] and, in the low-affinity range, other transporters such as AMT2-1 come into play to contribute to ammonium transport [30]. Ammonium transporters are well studied in rice. This species contains 10 members of the AMT family, three OsAMT1 family members characterized as high-affinity transporters, while three OsAMT2, three OsAMT3, and one OsAMT4 members are considered low-affinity transporters [31]. Genes encoding these transporter proteins display different expression patterns; some are constitutively expressed in roots or shoots and others are up-regulated after exposure to ammonium or by N deprivation [32,33,34].

Additionally, several studies have demonstrated that cereal crops are able to take up organic N compounds, especially in low N conditions [35,36].

### 2.2. Nitrogen Reduction and Assimilation

Once nitrate is incorporated in plant cells, it is reduced to nitrite, in a reaction that takes place in the cytosol and is catalyzed by a nitrate reductase (NR) [37]. Nitrite is then translocated to the plastids and chloroplasts where it is reduced to ammonium by the nitrite reductase enzyme (NiR). Ammonium deriving from nitrate, or the one produced by photorespiration or amino acid recycling, is mostly assimilated in the plastids by the GS/GOGAT cycle [13]. Glutamine synthase (GS) catalyzes the fixation of ammonium on a glutamate (Glu) molecule to produce glutamine (Gln). Subsequently, glutamine reacts with 2-oxoglutarate to produce two molecules of glutamate in a reaction catalyzed by the glutamate synthase (GOGAT).

While nitrate reduction is rarely limiting for biomass production or optimal grain yield in cereal crops, this is not the case for the ammonia assimilatory pathway [38]. Nitrogen assimilation is an important metabolic step that can regulate grain yield and NUE. Several gene manipulation studies focused on the overexpression of glutamine synthetase (GS)/glutamate synthase (GOGAT) cycle genes have demonstrated it. These studies showed an enhanced growth rate, yield, and biomass in rice plants overexpressing *OsGS1* [39], (Table 1), and in transgenic wheat transformed with a GS gene from common bean [40]. In wheat, increased GS1 activity in leaves led to enhanced capacity to accumulate nitrogen, mainly in the grain, increasing grain dry matter. In rice, a small family of GS and GOGAT is present, however, the cytosolic GS1;2 and the plastidic NADH-GOGAT1 are responsible for the primary assimilation of ammonium ions in the roots. Overexpression of *GS1* gene in this species showed an improvement in N harvest index and N utilization efficiency, but no change was observed in NUE under a N-deficient environment as compared with standard N conditions.

Maize carries the NAD–malic enzyme type of C_4_ photosynthetic plants; this is the reason why it has a better capacity to assimilate and metabolize carbon (C) and N compounds as compared with plants undergoing C_3_ photosynthesis. Overexpression of the *Gln1-3* and *Gln1-4* genes in maize leads to an increase of kernel number [41] (Table 1), while their knockout mutations produce a reduction of kernel size [42], providing evidence that these genes may play a major role in kernel yield (Table 1). Moreover, the analysis of sorghum *Gln1* overexpressing lines showed a higher accumulation of the *Gln1* transcripts and up to 2.2-fold higher GS activity as compared with the nontransgenic controls [43]. Finally, in a recent study, the transformation of barley plants using a cisgenic strategy to express an extra copy of native *HvGS1-1* led to increased *HvGS1-1* expression and GS1 enzyme activity; overexpressing lines displayed higher grain yield and NUE as compared with wild-type plants [44] (Table 1).

### 2.3. Nitrogen Translocation and Remobilization

Asparagine is the major translocated amino acid in crops such as pea; whereas in cereals, similarly to tomato and tobacco, Gln is the preferentially exported N-compound. However, it has been demonstrated that all plants (monocotyledonous, dicotyledonous, C3 or C4 photosynthesis types) share common N remobilization mechanisms [13].

During senescence, asparagine and glutamine concentrations increase in the phloem sap and both amino acids are likely to play an important role in making N available in the senescing leaf for remobilization to the reproductive organs. In fact, *AsnS1* genes, encoding for asparagine synthetase, seem to play a role in N remobilization in flag leaves during grain filling in durum wheat [45]. In rice, approximately 80% of the nitrogen of the panicle is derived from senescing organs and reaches the reproductive organs through the phloem. Since Gln is the major form of N in the phloem sap, GS and GOGAT enzymes are important for N remobilization and reutilization in senescing and developing organs, respectively [27]. Several studies have shown that GS1-1 is responsible for this process and that NADH-GOGAT1 has an important role in the reutilization of transported Gln in rice developing organs [27,46]. In maize, wheat, and barley, the grain N content is correlated with flag leaf senescence, which seems to play an important role in N availability for grain filling [42,47]. It has been demonstrated that leaf senescence is essential also for yield. A delay in leaf senescence produces prolonged photosynthesis, which increases grain yield. However, delaying leaf senescence also decreases N remobilization efficiency and the grain protein content [13].

## 3. Transcription Factors Regulating N Use in Cereals

Transcription factors (TFs) operate as major switches in plant regulatory networks [48,49] and have proven to be involved in nitrogen use. In Arabidopsis, ANR1, belonging to the MADS box TFs, was discovered to regulate the production of lateral roots in response to nitrate [50] and to be implicated in the signaling pathway of NRT1.1 [51]. Several nitrate transporter genes are also regulated by members of the NLP (NIN-like protein) family of TFs [52,53]. Other TFs have been shown to interact with *NLP* genes, such as TCP20 (teosinte branched1/cycloidea/proliferating cell factor1-20; [54]), involved in the regulation of lateral roots in response to nitrate [55], and NRG2 (nitrate regulatory Gene2; [56]). Additional TFs involved in the regulation of N uptake have been discovered through system biology approaches and thanks to the boost of bioinformatics tools and computing power (for review see [5]). Through a machine-learning approach, the TFs *BT1* (bric-a-brac/tramtrack/broad) and *BT2* were found to act as hub genes in the network of nitrate response. Functional analysis indicated that these genes regulate traits determining NUE in Arabidopsis, and this regulation was also found for *BT* orthologues in rice, highlighting the aptness of Arabidopsis as a model plant for the study of regulatory networks in crop plants like cereals [57] (Table 1).

DOF (DNA-binding with one finger) transcription factors are involved in various biological processes, such as hormone signaling and tissue differentiation [58]. The transgenic expression of Z. *mays DOF1* (*ZmDOF1*) gene in rice increases N assimilation and plant growth under low-N conditions [59] (Table 1). The same gene was introduced also in bread wheat and sorghum with a reduction of biomass accumulation in both cases [60] (Table 1). Recently, the *OsDOF18* gene was demonstrated to control ammonium uptake by inducing the *AMT1*, *AMT2,* and *AMT3* ammonium transporter family members in rice roots [61] (Table 1). A transcriptomic analysis of durum wheat adult plants exposed to nitrogen starvation displayed an overexpression of the *DOF1.3* gene in the roots of the stressed plants as compared with the controls [62]. The same work identified 170 unique genes encoding transcription factors belonging to different families, including *MYB*, *bHLH*, *bZIP*, *WRKY*, *mTERF*, *NAC*, *C2C2-Dof*, *NF-Y*, the auxin-modulated *ARF* and *AUX/IAA*, etc., which display differential expression between N stressed and control durum wheat tissues. Many of the above genes, as well as other TFs, have been detected as target genes of miRNAs (see below).

## 4. MiRNAs and Target Genes Involved in the Regulation of N Use in Cereals

A number of studies based on massive sequencing or computational analyses have been carried out in cereal crops highlighting miRNAs involved in the response to N deficiency. In rice, a comprehensive miRNA expression profiling in plants exposed to different N sources and treatments revealed a vast difference in miRNA response to nitrate and ammonium treatments, providing information on the regulation of N signaling and homeostasis mediated by miRNAs [63]. In a recent study, rice N-starved roots and shoots were subjected to a combination of multiple RNA sequencing analyses to widely describe the expression of genes, miRNAs, and lncRNAs (long non-coding RNAs), providing a deep insight into the regulatory pathways modulated by miRNAs responding to N starvation [64]. In shoots, most of the differentially expressed miRNAs were up-regulated in seven-day N-starved rice plants. Conversely, in roots, the majority of differentially expressed miRNAs were down-regulated in seven-day N-starved plants, and a significant down-regulation in response to N starvation was observed for the members of the osa-miR169 family [64]. In maize, a microarray analysis allowed the identification of miRNA families in leaves (nine families) and roots (nine families) responding to chronic or transient nitrate deficiency, showing overlapping or unique responses to different NO_3_^−^ limitation, as well as tissue specificity [65]. A study under N deficiency and resupply in maize highlighted that over 100 conserved miRNAs were differentially expressed (DE) between the control and N-deficient plants, and about 100 miRNAs were DE between the N-deficient and N-resupply groups of plants. In particular, miR169, miR1214, miR2199, miR398, miR408, and miR827 families might participate in the regulation of nitrogen metabolism in maize [66].

In durum wheat, following a chronic N deficiency experiment, eight small RNA libraries were produced using leaves and roots from two varieties showing a contrasting use of N (Ciccio and Svevo, [67]). Quantitative PCR of conserved and novel miRNAs highlighted that the expression levels of some miRNAs depended on the tissue and on the cultivar. Moreover, a difference in miRNA expression was also observed in durum wheat plants subjected to chronic N stress, as compared to plants that underwent a short-term N deprivation [68] (Table 1, Table 2, and Table S1).

A computational-based study in barley has found 156 mature miRNAs belonging to 50 miRNA families. Many previously known and several putatively new miRNA/target pairs were found. The identified miRNAs are related to development and response to biotic and abiotic stress and most of the target genes are involved in transcription regulation [69]. Additionally, putative polymorphisms in the miRNA target sites were identified in the same work.

Several miRNAs with important roles in mediating plant tolerance to N-starvation stress and their target genes have been identified also in wheat species. Analysis of miRNA expression in root tissues of several wheat genotypes showed that miR159a, miR159b, miR399, and miR408 are differentially expressed in response to N availability and wheat genotypes [70] (Table 1).

Several miRNAs play a key role in the regulation of nitrogen stress adaptation in cereal crops modulating the expression of genes involved in N uptake and remobilization.

### 4.1. MiRNA Regulation of N Uptake

Members of the miR169 family are reported to regulate the expression of key target nitrogen transporters under N deprivation conditions. In Arabidopsis, miR169 targets *NFY* (Nuclear Factor Y), a ubiquitous transcription factor, which binds the promoter regions and regulates nitrate transporters AtNRT2.1/AtNRT1.1 [71]. Under N deficiency, there is a strong down-regulation of miR169, while its target *NFYA* family members are strongly induced in both roots and shoots. Moreover, miR169a overexpression represses *NFYA* expression and produces a hypersensitivity to N starvation in the plants associated with the down-regulation of the NRT2.1 and NRT1.1 nitrate transporter genes, which suggests an important role of miR169 in N uptake and remobilization [71].

**Figure 1:**
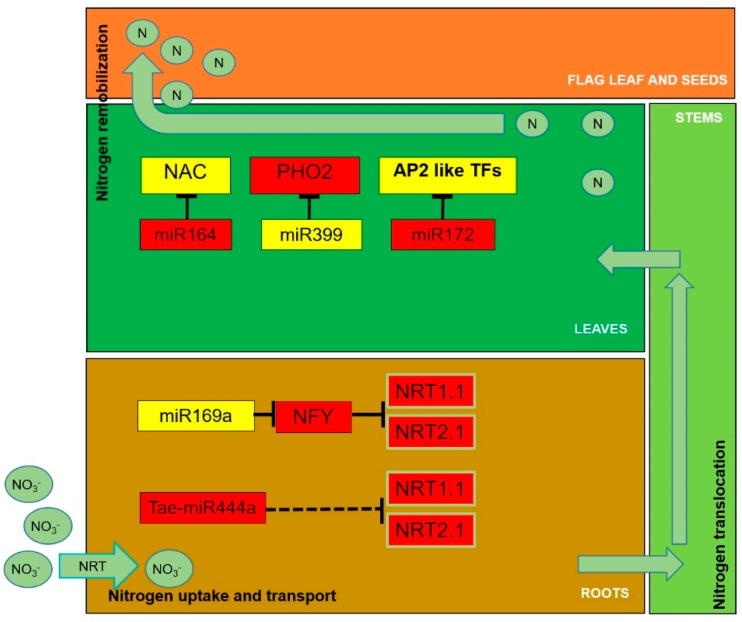
An example of the regulation operated by some miRNAs on genes involved in nitrogen use in cereals. Arrows indicate nitrogen (in various forms) transport. Red: up-regulation; yellow: down-regulation; TF: transcription factor.

Members of the miR169 family have also been studied in cereal crops. In maize, miR169 expression clearly decreases in N-deficient plants [72] (Table 2, Table S1) and an up-regulation of *NFYA* genes is observed in bread wheat plants growing in nitrogen starvation environments [73] (Figure 1). Overexpression of *TaNFYA-B1* in soft wheat activates lateral branching, nitrate transporter expression, increasing N uptake and grain yields in N-deficient conditions [73] (Table 1). Furthermore, our research group identified several conserved and new miR169 family members responding to nitrogen starvation environments in durum wheat [67,68] (Table 2, Table S1). The conserved ttu-miR169h at the seedling stage, ttu-miR169c at the grain filling stage, and the newly identified ttu-novel-61 belonging to the miR169 family, were down-regulated in both stages of durum wheat plants submitted to N starvation in both roots and leaves. Moreover, ttu-novel-61 was shown to negatively regulate *WHAP6*, a CCAAT box-binding transcription factor (corresponding to the *NFY* gene) in several tissues of durum wheat plants [67]. According to these results and comparing them with the literature, *CCAAT-TF WHAP6* could be an activator of nitrogen transport and therefore a candidate gene for potential genetic improvement programs aimed at increasing the yield of grain with less use of fertilizers in durum wheat.

Deep sequencing of small RNA and degradome libraries of maize seedlings submitted to N deficiency revealed 99 loci belonging to 47 miRNA families, nine of these paralogs of miR169, miR171, and miR398 [72] (Table S1). Additionally, eight miRNA families were differentially expressed under N-deficient conditions and target analysis suggested a role of the newly identified miRNA target genes in a broad range of cellular responses and metabolic processes [72]. More recently, another small RNA and degradome sequencing study together with target gene validation showed that two novel putative miR169 species, miRC10- and miRC68, may play a major role in the adaptation to low nitrogen in maize seedlings [74]. In maize roots, miR169i/j/k, miR169i*/j*/k* were repressed in response to low NO_3_^−^, suggesting an important role of these miRNAs in integrating NO_3_^−^ signals into root developmental changes [75] (Table 2 and Table S1).

Overexpression of TaMIR444a in tobacco modifies the transcriptome and improves plant growth and dry mass production under N deprivation. Expression analysis of transgenic plants showed an up-regulation of the tobacco *NtNRT1.1-1*, *NtNRT1.1-2*, and *NtNRT2.1-1* genes, suggesting that TaMIR444a regulates N acquisition through the modulation of *NRT* genes [76] (Figure 1, Table 1 and Table 2, and Table S1). Moreover, a recent transcriptome analysis in rice highlighted a down-regulation of a root-specific miRNA, osa-miR444a.4-3p, in N starvation conditions and its target gene *MADS25* was confirmed by degradome sequencing [64] (Table 2, Table S1).

### 4.2. MiRNA Regulation of N Remobilization

In Arabidopsis, miR164 guides the cleavage of endogenous and transgenic *NAC1* mRNA. Additionally, it has been shown that *mir164a* and *mir164b* mutants expressed less miR164 and more *NAC1*, as compared with wild-type plants [77]. Several works have aimed at the characterization of *NAC* genes and miR164 family members in cereal crops (Table 2, Table S1). In bread wheat, it is known that the *NAM-B1* gene is a NAC transcription factor affecting grain nutrient concentrations [78] and increasing nutrient remobilization from leaves to developing grains in ancestral wild wheat [48]. Additionally, in maize, zma-miR164 is down-regulated in leaves after a chronic N stress treatment [65]. It is suggested that, in cereals, regulation of *NAC* genes by miR164 may occur to maintain the remobilization of N from leaves to seeds in low N conditions (Figure 1). Microarray-based miRNA expression analysis, in low-N tolerant and sensitive rice genotypes under low-N conditions, showed 32 miRNAs differentially expressed between the two genotypes including miR164 and another seven miRNAs. Six miRNAs were differentially expressed in leaves (miR164, miR156, miR528, miR820, miR821, and miR1318) and four in roots (miR164, miR167, miR168, and miR528) [79]. The miRNAs identified were predicted to regulate genes encoding transcription factors and proteins associated with metabolic processes or stress responses.

### 4.3. Other N Stress Responsive miRNAs

In the roots of maize, miR528a,b and miR528a*,b* were repressed in response to low NO_3_^−^, suggesting a role of these miR528 family members in integrating NO_3_^−^ signals into root developmental changes [75] (Table 2 and Table S1). Additionally, Zma-miR528a,b family members were down-regulated in maize leaves and roots of seedlings exposed to N deficiency [72] (Table 2 and Table S1). Another recently published work reveals that the expression of Zma-miR528, a monocot-specific miRNA, is reduced by N deficiency and induced by N luxury, regulating the expression of two genes encoding for copper-containing laccases (*ZmLAC3* and *ZmLAC5*) [41] (Table 1). Rice miR528 was expressed in *Arostis stolonifera*, with a subsequent increase in plant biomass, total N accumulation, and chlorophyll synthesis [80] (Table 1).

In addition to miR528, another expression and functional study identified Osa-miR393 as a regulator of *OsAFB2* and *OsTB1* genes in rice; moreover, miR528 was shown to be involved in N-mediated tillering by decreasing auxin signal sensitivity in axillary buds [81] (Table 1). In common wheat, TaMIR1118, TaMIR1129, and TaMIR1136 were up-regulated, whereas TaMIR1133 was down-regulated in roots, in N-deprived conditions. The expression of some of these miRNAs was inversely correlated with N concentration and low-N duration [82] (Table 2 and Table S1). Another common wheat miRNA, TaMIR2275, was gradually up-regulated during N starvation, while its expression was progressively restored upon N recovery treatment. Overexpression of this miRNA produced plants with increased biomass and N accumulation, while the opposite was observed for knockdown mutants. It was suggested that TaMIR2275 is crucial in plant N stress response through transcriptional regulation of target genes involved in N acquisition [83] (Table 1, Table 2, and Table S1).

**Table 1:** MiRNAs and genes involved in nitrogen use in cereals, which have been functionally validated through a transgenic approach or mutations. Prefix of gene or miRNA name indicates the plant species (e.g., Os: *Oryza sativa*). References are numbered as in the reference list.

miRNA/Gene	Species Transformed	Genetic Modification	Genes Functionally Validated as miRNA Targets	Effects of the Transgenic/Knockout Gene or miRNA	Reference
Osa-miR393	*O. sativa*	Overexpression and Knockout mutation	OsAFB2 and OsTB1	Overexpression mimicked N-mediated tillering and knockout mutation repressed N-promoted tillering	[81]
OsDof18		Knockout mutation		Reduction of the expression of ammonium transporter genes and ammonium uptake	[61]
Osa-miR528	*A. stolonifera*	Transgenic expression	AAO, COPPER ION BINDING PROTEIN1	Increasing of biomass, total N accumulation and chlorophyll synthesis, nitrite reductase activity and reduced AAO activity	[80]
Zma-miR528		Knockdown mutation	ZmLACCASE3 (ZmLAC3) and ZmLACCASE5 (ZmLAC5)	Significant increasing of lignin content and rind penetrometer resistance of maize stems	[41]
ZmLAC3	*Z. mays*	Overexpression		Significant increasing of lignin content and rind penetrometer resistance of maize stems	[41]
OsGS1	*O. sativa*	Overexpression		Improving of N use efficiency	[80]
HvGS1-1	*H. vulgare*	Overexpression		Higher grain yields and NUE when grown under three different N supplies and two levels of atmospheric CO2. Improving of grain yield and NUE	[44]
Tae-MIR444a	*N. tabacum*	Transgenic expression	NtNRT1.1-s, NtNET1.1-t, NtNRT2.1 and AEEs; NtCAT1;1, NtPOD1;3, and NtPOD4	Increasing of N acquisition and cellular ROS detoxification in N-deprived plants	[76]
ZmDof1	*T. aestivum*	Transgenic expression		Increasing biomass and yield. Down-regulation of genes involved in photosynthesis	[60]
Zma-miR528	*Z. mays*	Overexpression	ZmLAC3 and ZmLAC5	Reduction of lignin biosynthesis under Nitrogen-Luxury Conditions	[41]
ZmGln1-3/ZmGln1-4		Knockout mutation		Reduction of kernel size and kernel number	[42]
SbGln1	*S. bicolor*	Overexpression		Greater tillering and up to 2.1-fold increase in shoot vegetative biomass under optimal nitrogen conditions	[43]
ZmDof1	*O. sativa*	Transgenic expression		Increasing of nitrogen assimilation and enhancing plant growth under low-nitrogen conditions	[59]
ZmDof1	*S. bicolor*	Transgenic expression		Increasing biomass and yield. Down-regulation of genes involved in photosynthesis	[60]
Tae-miR2275	*N. tabacum*	Transgenic expression and knockdown	TaPRP, TaBDP, TaWRK, TaSPK, TaPP, TaAAT, TaNTA, TaIM	Increasing of the biomass and N accumulation in overexpressing lines. Decreased biomass and plant N amount after N starvation in knockdown mutants	[83]
OsNRT2.3b	*O. sativa*	Overexpression		Increasing of N, Fe, and P uptake. Improving of the grain yield and nitrogen use efficiency (NUE) by 40%	[25]
OsBT	*O. sativa*	Mutation		Increasing of NUE by 20% under low nitrogen conditions	[57]
TaNFYA-B1	*T. aestivum*	Overexpression		Significant increasing of both nitrogen and phosphorus uptake and grain yield under differing nitrogen and phosphorus supply levels	[73]

## 5. MiRNAs and Crosstalk Between Nutrients in Cereals

Nutrient concentrations are not always sufficient for plant growth in agricultural soil. In order to adapt to these conditions, the metabolisms of different nutrients are interconnected so that when a nutrient is missing, the metabolisms of other mineral nutrients will be adjusted to maintain appropriate growth and development. Carbon and N are essential for plants to perform vital cellular activities, therefore, the adequate supply of these two nutrients is essential for plant growth, development, and response to the different environmental stresses. A balance between N and C, more than just one of these minerals, affects global gene expression [84].

After N, phosphorus (P) is the second most limiting nutrient in natural conditions and as is nitrogen, it is necessary for crop development and yield. Phosphorus is assimilated by plants as orthophosphate (Pi) and is a component of several biomolecules such as phospholipids from membranes, intermediates of photosynthesis and respiration, and nucleotides from nucleic acids and from the energy compounds ATP and GTP [85]. The addition of Pi to proteins affects their activity, and therefore P is a key player on protein regulation and signal transduction.

In Arabidopsis, an intimate crosstalk between N response and Pi pathways at the level of regulated proteolysis has been uncovered [86]. MiRNA regulation of the use of nitrogen and the crosstalk with P has been studied in more details in the model plant than in crops. Studies in Arabidopsis plants growing in N limitation conditions showed that the *nitrogen limitation adaptation* (*NLA*) gene is an essential component for developing the nitrogen limitation adaptive responses [87,88]. Moreover, *NLA* interacts with *PHO2/UBC24* gene, which encodes the ubiquitin-conjugating E2 enzyme 24. E2 and E3 pair together polyubiquitinates, the high-affinity Pi transporter PT2, for degradation [87]. *NLA* has also been demonstrated to be targeted by miR827, playing a pivotal role in regulating Pi homeostasis in a nitrate-dependent fashion [89]. During a Pi starvation response, NLA and PHO2 transcripts are cleaved by miR827 and miR399, respectively, releasing the posttranslational repression of PT2, thus, allowing this protein to accumulate and participate in Pi uptake [90,91,92]. Observations of Arabidopsis transgenic plants overexpressing miR399 and *pho2* mutants show that, as a consequence of a high Pi uptake, a lack of *PHO2* produces Pi toxic symptoms in shoots, Pi translocation from roots, and Pi retention in old leaves [91,93]. A Pi starvation environment triggers the up-regulation of *AT4* and *IPSI* factors that mimicry *PHO2* target sequence and as a consequence of this, there is an up-regulation of miR399 [94]. *PHO2* gene has an important role in the maintenance of phosphate homeostasis, demonstrating the profound interconnections between the processes concerning the various plant nutrients.

In durum wheat, miR399b was strongly inhibited under stress conditions; on the contrary, as expected, its target *PHO2* gene was strongly activated in the same tissues, suggesting a similar role as in Arabidopsis [68] (Figure 1, Table 1, Table 2, and Table S1). In rice, miR399 was up-regulated in plantlets after N stress [95] (Table 2 and Table S1). In a gene regulatory network analysis in response to N in maize, it was observed that miR399b interacted with 97 other nodes in the network, suggesting that it is a crucial regulator related to N metabolism [96].

## 6. Conclusions

Cereals are the main source of food worldwide, with increasing cultivation especially for maize, wheat, and rice, which are the most consumed cereal crops. Vitality and production of cereal crops depend on the availability of N, which has led to the huge production and use of N fertilizers worldwide, with several negative consequences on the environment and human health. A better understanding of the regulation of N use in cereals opens perspectives for obtaining plants with a higher NUE, and thus improved sustainable agriculture. MiRNAs play a pivotal role in this challenge, since they have proven to be key regulators of genes, some of which are involved in cereal NUE.

**Table 2:** Conserved miRNAs responsive to nitrogen deficiency in rice, maize, and wheat and their target genes. The miRNAs here listed have been validated through various techniques. References are numbered as in the reference list. L: leaves; R: roots.

miRNA Families		Rice		Maize		Bread Wheat		Durum Wheat		Validated/Putative Target Genes	Reference
		L	R	L	R	L	R	L	R		
miR156										Squamosa promoter binding protein-like (SBP-box)	[72,79,82]
miR157				*							[66]
miR159										MYB33, MYB65	[63,72]
miR160										Auxin response, ARF22	[65,68,70]
miR162										DCL1	[72]
miR164									^	NAC, NAC7	[65,68,70,72,79]
miR166										START domain containing protein, HD-Zip TFs	[68,75]
miR167								^	^	ARF8	[65,67,68,72,79]
miR168										ARGONAUTE1	[65,79]
miR169				*						CCAAT-TF WHAP6, HAP2 like protein	[63,65,66,67,72,73,75]
miR171										Scarecrow-like TF; Protein FAN	[72]
miR172										AP2 like TFs, APETALA2, Bzip TF family protein	[63,65,72]
miR319								^		MYB and TCP transcriptional factors	[65,67]
miR393										AFB2	[67,72]
miR394										F-box domain containing protein	[72]
miR395										APS1, APS4	[65,72]
miR396										GRF TFs, rhodenase-like proteins, kinesin-like protein B	[72]
miR397				*						Laccase	[65,66,72]
miR398				*						COX	[65,66,72]
miR399										PHO2	[65,67,68,70,72,82,95]
miR408				*						PLANTACYANIN	[65,66,72,75]
miR415				*						Aminoacylase; N-acyl-L-amino-acid amidohydrolase	[66]
miR444									^	MIKC-type MADS-box TFs, Maturase K, GRAS TFs	[64,67,68,76,82]
miR528										IAR1, CBP/OsDCL1, POD, SOD	[41,65,72,75,79]
miR529										Squamosa promoter binding protein-like (SBP-box)	[63]
miR530										Hairpin-induced protein 1 domain containing protein	[95]
miR820										DRM2 (DNA (cytosine-5)-methyltransferase)	[79]
miR821										GDH1 (Glutamate dehydrogenase)	[79]
miR827				*				^		SPX E3 ligase, CLP	[65,66,67,68]
miR1118											[82]
miR1129											[82]
miR1133										Calmodulin-like, SET domain, early nodulin proteins, etc.	[82]
miR1136											[82]
miR1214				*							[66]
miR1318										Calcium binding proteins or Calcium ATPases	[79]
miR2199				*							[66]
miR2275						*				PRP, BDP, WRK, SPK, PP, AAT, NTA, IM	[83]
miR3979											[63]
	Down-regulated										
	Up-regulated										
	Different miRNA family members display different expression pattern										
	Different developmental stages display different expression pattern										
*	Seedlings										
^	Different behaivor in different crop varieties										

## Supplementary Materials

The following are available online at https://www.mdpi.com/2223-7747/8/8/294/s1. Table S1: Nitrogen responsive miRNAs in rice, maize, and wheat. The miRNAs listed in the table have been experimentally validated. Highlighted in pink are the miRNAs with different expression behavior due to the different duration of the N stress treatment. References are numbered as in the reference list.
